# Biomechanics of sacroiliac joint fixation using lag screws: a cadaveric study

**DOI:** 10.1186/s13018-023-04311-5

**Published:** 2023-10-28

**Authors:** Grégoire P. Chatain, Alton Oldham, Juan Uribe, Bradley Duhon, Michael J. Gardner, Jens-Peter Witt, Scott Yerby, Brian P. Kelly

**Affiliations:** 1 Department of Neurosurgery, University of Colorado School of Medicine, 12605 E 16Th Ave, Aurora, CO 80045 USA; 2 Spinal Biomechanics Laboratory, Department of Neurosurgery, Barrow Neurological Institute, St. Joseph’s Hospital and Medical Center, Phoenix, AZ USA; 3 Department of Neurosurgery, Barrow Neurological Institute, St. Joseph’s Hospital and Medical Center, Phoenix, AZ USA; 4 Department of Orthopaedic Surgery, Stanford University School of Medicine, Redwood City, CA USA; 5 SI-BONE, Santa Clara, CA USA

**Keywords:** Biomechanics, Sacroiliac joint, Lag screw, Fusion, Trauma

## Abstract

**Background:**

Iliosacral screw placement is ubiquitous and now part of the surgeon’s pelvic trauma armamentarium. More recent evidence supports sacroiliac arthrodesis for treating sacroiliac joint (SIJ) dysfunction in select patients. Regardless of the surgical indication, there are currently no studies examining lag screw compression biomechanics across the SIJ. The objective of this biomechanical investigation was to quantify iliosacral implant compressive loads and to examine the insertion torque and compressive load profile over time.

**Methods:**

Eight human cadaveric pelvic specimens underwent SIJ fixation at S1 and S2 using 11.5 and 10.0 mm iFuse-TORQ Lag implants, respectively, and standard 7.3 mm trauma lag screws. Load decay analysis was performed, and insertion and removal torques were measured.

**Results:**

For both implants at S1 and S2 levels, the load relaxed 50% in approximately 67 min. Compressive load decay was approximately 70% on average occurring approximately 15 h post-insertion. Average insertion torque for the 11.5 mm TORQ implant at S1 was significantly greater than the trauma lag screw. Similarly, at S2, insertion torque of the 10.0 mm TORQ implant was greater than the trauma lag screw. At S1, removal torque for the 11.5 mm TORQ implant was higher than the trauma lag screw; there was no significant difference in the removal torque at S2.

**Conclusions:**

In this study, we found that a novel posterior pelvic implant with a larger diameter, roughened surface, and dual pitch threads achieved improved insertion and removal torques compared to a standard screw. Load relaxation characteristics were similar between all implants.

## Introduction

Sacroiliac joint (SIJ) hypermobility or aberrant biomechanics is a common source of low-back pain. It is estimated that SIJ dysfunction is the underlying etiology for 15–30% of patients suffering from chronic low-back pain [1–3]. Randomized clinical trials support sacroiliac arthrodesis for treating SIJ dysfunction in carefully selected patients who previously failed conservative measures [4–8]. This growing body of the literature demonstrates that SIJ fusion (SIJF) leads to improvement in clinical outcomes as compared to those patients who were treated conservatively; it has been shown to be an effective way of reducing pain, increasing quality of life and reducing opioid use [5, 7, 9]. Additionally, traumatic disruption of the SI joint is frequently treated with reduction and screw fixation, but patient outcomes are unpredictable and are often associated with long-term SIJ pain [10].

The emergence of minimally invasive approaches (MIS) has allowed surgeons to safely and effectively treat SIJ dysfunction [4–8]. While limiting the morbidity associated with open approaches, MIS techniques result in improvements in Oswestry Disability Index scores. As a result, MIS approaches have gained traction over the last two decades. One advance has been the implantation of porous triangular titanium implants (iFuse system, SI-BONE) across the joint facilitating fusion without the need for bone grafting. Other studies also report on the use of screw-type implants for SIJF [11].

There may be a biomechanical benefit in using lag screws across the SIJ, which could potentially lead to improved stabilization and fusion of the joint [12, 13]. In a traumatic setting, the posterior pelvic injury frequently includes ligamentous disruption and diastasis of the SI joint. In this situation, lag or compression screws are necessary to reduce, compress, and stabilize the SI joint [14]. When the posterior pelvic injury is an unstable sacral fracture, surgeons often rely on percutaneous screw insertion with compression to achieve fracture surface apposition through reduction and increased stability [11, 15]. The terminally threaded screw design creates a lag effect, which reduces the fracture gap and reapproximates the fragments along the fracture line. Overall, lag screws are useful to achieve fracture reduction as well as interfragmentary compression, a fundamental requirement for fracture healing [16–18].

There are currently no studies investigating the biomechanical compression performance of lag screws over time across the SIJ. The objective of this biomechanical investigation was to quantify the immediate peak value and time course of compressive loads attained with iliosacral implants placed across the SIJ from the ilium to the mid-sacrum. Technological advances have allowed for additive manufacturing to create screws with a roughened porous layer that may confer both biomechanical, and potentially long-term biological advantages promoting bony ingrowth. We examined these biomechanical properties for both standard titanium trauma lag screw (DePuy Synthes, Raynham, MA) and titanium iFuse-TORQ screws with an underlying porous structure (SI-BONE, Santa Clara, CA). The implant diameters chosen for the current study represent commonly used diameters for a given sacral level; 7.3 mm standard titanium trauma lag screws are commonly used at each sacral level, and 11.5 mm additively manufactured implants are commonly used at the S1 level and 10.0 mm screws at the S2 level. The investigators hypothesized that lag screws would provide compression across the implants immediately following placement through the ilium, across the SI joint and into the mid-sacrum, but that this compressive load would considerably decay over time when compared to a larger diameter screw with a roughened surface. This study aimed to characterize and quantify these loading attributes over time.

## Methods

### Specimen preparation

We used eight fresh-frozen human cadaveric pelvic specimens with intact sacroiliac and pubic symphysis joints. All specimens were radiographed in multiple planes to confirm the absence of any significant osseous pathologies. Review of past medical records, bone mineral density studies (BMD), and direct visual inspections did not reveal any pathology or sacroiliac abnormality that could have altered the results. Specimens were thawed to room temperature in a saline bath (0.9% NaCl), and the paravertebral and pelvic musculature was carefully removed avoiding disruption of pertinent osteoligamentous structures especially around the SIJ. Lumbar dual-energy X-ray absorptiometry scans were performed to evaluate bone mineral density (g/cm^2^) on all specimens (Discovery W, Hologic Inc., Marlborough, MA).

### Sacroiliac joint screw fixation

**Fig. 1:**
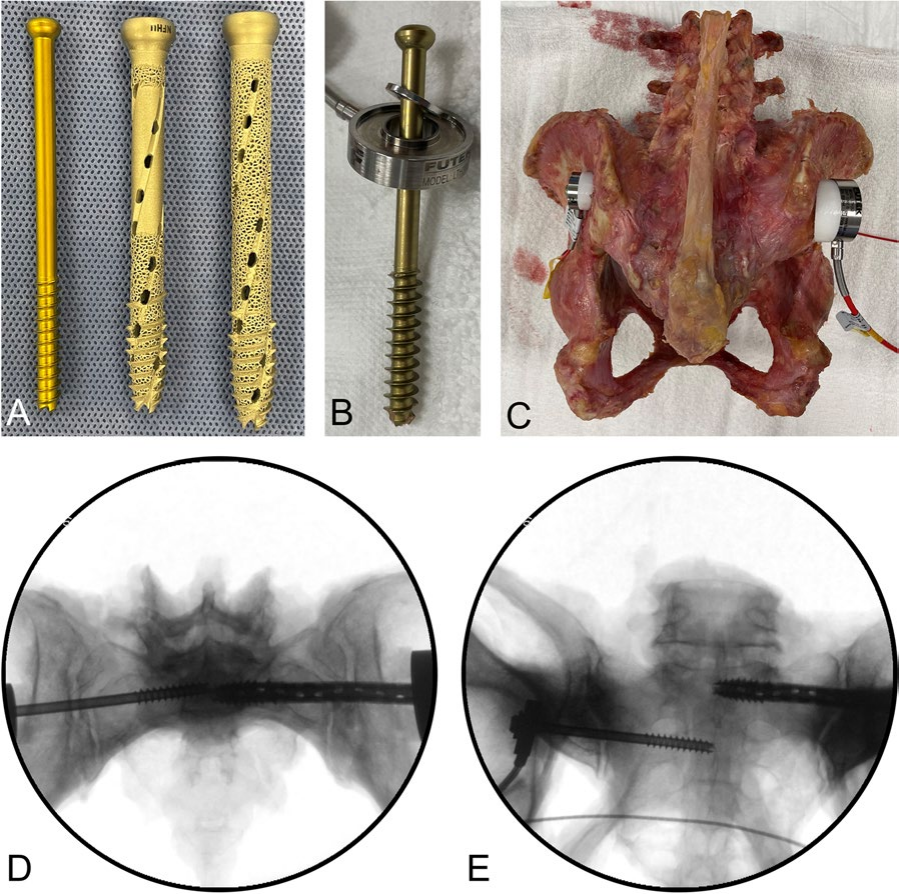
**a** The 7.3 mm (left), 10.0 mm Lag (middle), and 11.5 mm Lag implants. **b** The 7.3 mm implant and one of the washer load cells. **c** A specimen with a load cell and wedge washer (white) on the left and right sides. **d** An inlet view of a 11.5 mm implant placed at S1 and a 7.3 mm implant placed at S2. **e** An outlet view of a 11.5 mm implant placed at S1 and a 7.3 mm implant placed at S2

For SIJ fixation, screws were placed under fluoroscopic guidance using a guide K-wire inserted through the bony corridor of the sacral ala into the body of the S1 or S2 vertebrae. A cannulated drill bit was placed over the guide wire and used to go through the medial cortex of the ilium for all TORQ implants. All TORQ screw tracks were subsequently tapped, while the trauma lag screws were self-tapping. Radiographs of SIJ screw placements were performed on lateral, anteroposterior, inlet, and outlet views to rule out breaches and confirming screw tip insertion to midline (Fig. 1); this was also confirmed with direct visualization and palpation since the musculature had been removed. Screws were placed to midline to allow for a paired comparison of left and right implants placed at both S1 and S2.

We placed two differently sized iFuse-TORQ lag screws (SI-BONE, Santa Clara, CA) and paired them with conventional trauma lag screws (DePuy Synthes, Raynham, MA); 11.5-mm-diameter iFuse-TORQ implants (11.5 screw) and 7.3-mm-diameter trauma lag screws (7.3 screw) were placed at the S1 level and 10.0-mm-diameter iFuse-TORQ implants (10 screw) and 7.3-mm-diameter trauma lag screws were placed at the S2 level. The standard 7.3-mm-diameter screws had a pitch of 2.75 mm/thread, a core diameter of 4.5 mm, an outer diameter of 7.3 mm, and a buttress thread profile. The 10.0 mm screw had a pitch of 5.5 mm/thread, a core diameter of 7.5 mm, an outer diameter of 10 mm, and a reverse buttress thread profile (Fig. 1). The 11.5 mm screw had a pitch of 5.5 mm/thread, a core diameter of 9 mm, an outer diameter of 11.5 mm, and a reverse buttress thread profile. The 7.3 mm screws were smooth, and the 10 and 11.5 mm screws were rough with pores and fenestrations (Fig. 1). Both types of implants were placed in each specimen, and sides were alternated, which allowed for a paired analysis. The bony corridor above the S1 foramen can often accommodate a larger implant diameter than the corridor between the S1 and S2 foramina, and therefore, an 11.5-mm-diameter implant was placed at S1 and a 10.0-mm-diameter implant was placed at S2.

Data were acquired with an S1 screw placed on one side, while an S2 screw was placed on the contralateral side. Sides were alternated at the end of data acquisition for a total of 4 SIJ screw placements per specimen; the left–right alternating screw placement reduced bias of one given treatment placed only on one specimen side. All implants were placed by a trained neurosurgery resident (GC). A pilot study using all tested implants and seven pelvic cadaver specimens preceded this study to improve the likelihood that all final specimens had correct implant placement and tightening. During the trial, some of the implants were intentionally stripped to gain a better understanding of the tightening limits. In the study, the implants were tightened to the surgeon’s feel based on his trial experience. Attempts to quantify the insertion torque limit proved difficult during the trial based on factors such as specimen bone quality and morphometry.

Compressive load data were acquired for all implants at both S1 and S2 levels. Specifically, peak load (N), time to 50% load reduction (s), and percent load drop at steady state (%), which was defined as a relaxation load that did not change for a period of at least three minutes, were obtained (Table 1).

**Table 1:** Specimen demographics

Specimen	Gender	Age (yr)	BMD (g/cm^2^)
#1	M	61	0.951
#2	M	37	1.060
#3	M	58	0.969
#4	F	57	0.573
#5	F	57	0.953
#6	M	66	0.974
#7	F	67	0.701
#8	M	61	0.880

**Table 2:** Compression data for the different implants at S1 and S2

Treatment	Insertion torque (Nm)	Removal torque (Nm)	Peak load (N)	Time to 50% peak load (s)	Load drop at steady state (%)	Steady-state load (N)
7.3 S1	2.33 ± 1.35	1.49 ± 1.62	423.8 ± 300.4	971 ± 1013	69.5 ± 4.0	130.5 ± 88.7
7.3 S2	1.74 ± 1.50	0.94 ± 0.92	341.7 ± 301.5	1827 ± 3130	71.1 ± 7.7	97.8 ± 87.8
11.5 S1	5.04 ± 1.91	2.82 ± 1.13	212.6 ± 134.8	2100 ± 4984	70.1 ± 8.3	71.1 ± 59.7
10.0 S2	2.95 ± 1.58	0.94 ± 0.60	205.7 ± 112.7	12,545 ± 30,691	79.4 ± 14.8	44.9 ± 39.4
*p values*						
7.3 S1 vs 11.5 S1 (paired, *n* = 8)	**0.003**	**0.049**	0.081	0.568	0.855	0.125
7.3 S2 vs 10.0 S2 (paired, *n* = 6)	**0.005**	0.875	0.368	0.456	0.327	0.340
7.3 S1 vs 7.3 S2 (paired, *n* = 7)	**0.020**	**0.034**	0.086	0.306	0.351	0.080

Bold *p* values represent significant differences

### Data collection

Insertion and removal torque values were collected at a rate of 10 Hz using a torque sensor and controller (Model 01190-121, Sensor Development Inc., Orion, MI). Washer load cells (FUTEK Advanced Sensor Technology Inc., Irvine, CA) were used to measure the compressive load during and after instrumentation. Four load cells (model LTH300) with a capacity of 500 lbs (2224 N) and four load cells (model LTH350) with a capacity of 1000 lbs (4448 N) were used to accommodate the different implant diameters and to collect the compression loads for the lag trauma screws and the iFuse-TORQ screws, respectively (Fig. 1). Load cell data were collected using a StrainSmart data acquisition system (Vishay Micro-Measurements, Raleigh, NC). During instrumentation, and the two hours following implantation, load data were collected at a rate of 10 Hz to capture the rapid loading changes immediately after tightening. After the initial two hour initial data collection, the data collection rate was switched to 1/60 Hz for the remainder of the overnight test to capture the remaining compression load history.

### Statistical analysis

Data analysis was performed with Prism 9 (GraphPad Software, San Diego, CA). Two-tailed paired *t* tests were used to analyze the differences between screw compressive loads, as well as insertion and removal torques at each sacral level. Correlations between BMD and biomechanical findings such as insertion/removal torque and implant load were evaluated using correlations coefficients and t-scores. Statistical significance was defined as *p* < 0.05.

## Results

The average specimen age was 58 ± 9.3 years for 5 male and 3 female specimens (Table 1). The average bone mineral density (BMD) was 0.88 ± 0.15 g/cm^2^ (Table 1).

### Torque analysis of implants

All implants were successfully placed within the bony corridors above the S1 foramen for the S1 implants and between the S1 and S2 foramina for the S2 implants; this included the larger 11.5 mm implants at S1 and 10.0 mm implants at S2. All implants were implanted to the sacral midline. Due to the larger load cell size used for the 10.0 and 11.5 mm implants relative to the load cell used for the 7.3 mm implants, the median implant length used for the 10.0 and 11.5 mm implants was 10 mm longer than that of the 7.3 mm implants: 7.3 mm at S1 100 mm; 11.5 mmm at S1 110 mm; 7.3 mm at S2 90 mm; and 10.0 mm at S2 100 mm. This resulted in similar implant engagement within the pelvis for a given level, but different implant lengths within the load cells.

**Fig. 2:**
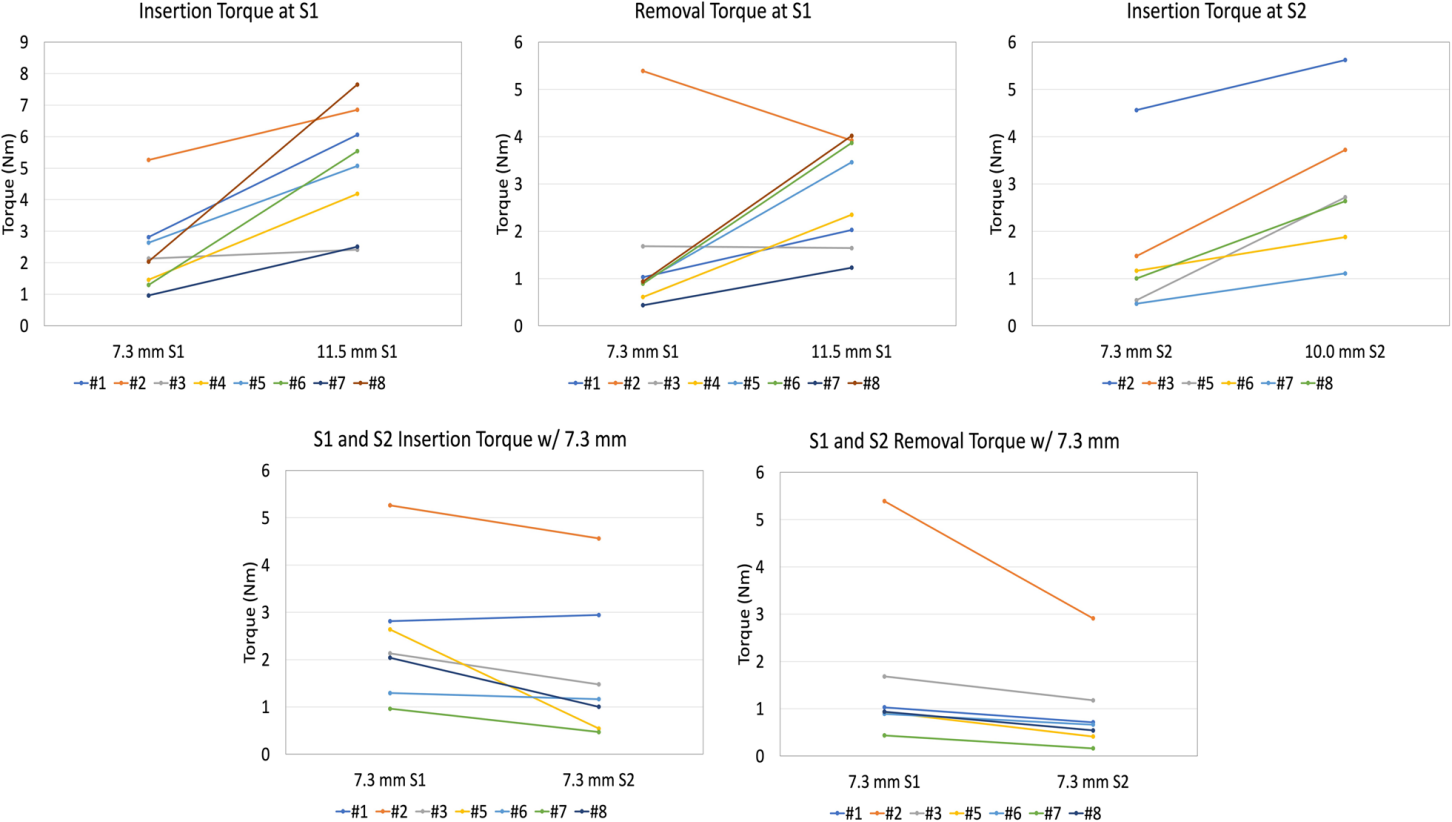
The individual insertion and removal torque values for the significant comparisons

Insertion and removal torques (Nm) were measured for all implants at both the S1 and S2 levels. During insertion, two of the iFuse-TORQ implants and one of the 7.3 mm implants stripped; all stripping occurred at the S2 level on two different specimens—one of which was Specimen 4 with the lowest BMD (0.573 g/cm^2^) in which the 10.0 mm and 7.3 mm implants stripped at the S2 level. Therefore, the paired analysis allowed for eight pairs at S1 and six at S2. The average insertion torque for the 11.5 mm TORQ implant at S1 (5.04 ± 1.91 Nm) was significantly greater than that of the trauma lag screw (2.33 ± 1.35 Nm) (*p* = 0.003, Fig. 2, Table 2). Similarly, at the S2 level, the insertion torque of the 10.0 mm TORQ implant (2.95 ± 1.58 Nm) was significantly greater than that of the trauma lag screw (1.74 ± 1.50 Nm) (*p* = 0.005, Fig. 2, Table 2).

At S1, the removal torque for the 11.5 mm TORQ implant (2.82 ± 1.13) was significantly greater than that of the trauma lag screw (1.49 ± 1.62 Nm) (*p* = 0.049, Fig. 2, Table 2). At the S2 level, however, no significant difference in removal torque was found between the 10.0 mm screws and 7.3 mm screws (*p* = 0.875, Table 2). There was a significant correlation between insertion and removal torque for a given level and a given implant: 7.3 mm at S1 *R*^2^ = 0.8381, *p* < 0.0015; 11.5 mm at S1 *R*^2^ = 0.6754, *p* < 0.0124; 7.3 mm at S2 *R*^2^ = 0.9814, *p* < 0.0002; and 10.0 mm at S2 *R*^2^ = 0.8449, *p* < 0.0096. The percent differences between the insertion and removal torque for a given level and a given implant were as follows: 7.3 mm at S1 36%; 11.5 mm at S1 44%; 7.3 mm at S2 36%; and 10.0 mm at S2 68%.

When comparing both anatomical levels, S1 and S2, using the same 7.3 mm trauma lag screw design, both the average insertion and removal torques were significantly different: insertion (2.33 ± 1.35 Nm at S1 vs 1.74 ± 1.50 Nm at S2, *p* = 0.020) and removal (1.490 ± 1.62 Nm at S1 vs 0.94 ± 0.92 Nm at S2, *p* = 0.034) (Fig. 2, Table 2).

### Load decay analysis

**Fig. 3:**
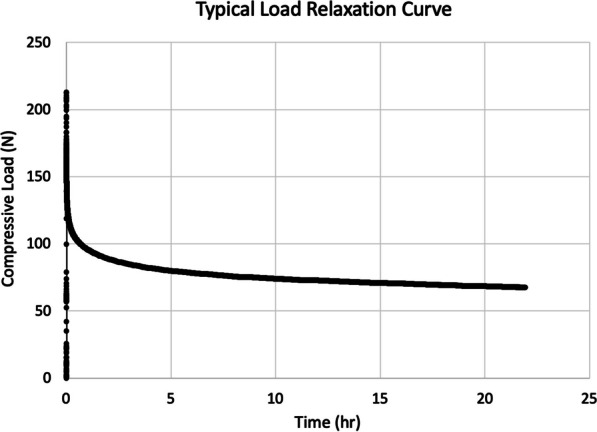
A representative load relaxation curve demonstrating the peak load at final implant tightening and the decay in load over time

Average peak load for all implants was 295.8 N (range 205.5–423.9 N) with no significance found between groups. When assessing all implants at both S1 and S2 levels, the load relaxed 50% in approximately 67 min on average (range 1 s–21 h); one second relaxation implants were stripped. Compressive load measurements over time revealed that the steady-state load decay was approximately 70% (range 69.6–79.4%) on average occurring in approximately 15 h after insertion (Fig. 3). No significant differences were found between implants and anatomical levels when evaluating the time to 50% peak load and the percent load drop at steady state (Table 2). There was a significant correlation between insertion torque and peak insertion load for a given level and a given implant: 7.3 mm at S1 *R*^2^ = 0.9750, *p* < 0.0001; 11.5 mm at S1 *R*^2^ = 0.5940, *p* < 0.0253; 7.3 mm at S2 *R*^2^ = 0.9570, *p* < 0.0008; and 10.0 mm at S2 *R*^2^ = 0.9521, *p* < 0.0010. There was no significant correlation between insertion torque and time to 50% peak load for a given level or implant. There was a significant correlation between insertion torque and steady-state load for the 7.3 mm implant at S1 (*R*^2^ = 0.9602, *p* < 0.0001) and the 7.3 mm at S2 (*R*^2^ = 0.9650, *p* < 0.0006), but not the 11.5 mm implant at S1 or the 10.0 mm implant at S2. There was no significant correlation between peak load and time to 50% peak load for a given level or implant. Finally, there was a significant correlation between peak load and steady state for a given level and implant with the exception of the 10.0 mm implant at S2: 7.3 mm at S1 *R*^2^ = 0.9878, *p* < 0.0001; 11.5 mm at S1 *R*^2^ = 0.9827, *p* < 0.0001; and 7.3 mm at S2 *R*^2^ = 0.9651, *p* < 0.0006.

### BMD correlations

There was no statistically significant correlation between BMD and any of the findings.

## Discussion

Percutaneous iliosacral screw placement has become widely popular and is now part of the surgeon’s armamentarium to treat unstable pelvic and sacral fractures. The technique has been refined over the past three decades with improved anatomic and fluoroscopic understanding, which has resulted in decreased morbidity [15, 19, 20]. More recently, evidence has supported sacroiliac arthrodesis for treating SIJ dysfunction in carefully selected patients [4–8]. Irrespective of the surgical indication, there are no prior studies that have clearly evaluated the biomechanics of lag screws across the SIJ pertaining specifically to compressive loads and relaxation rates.

In this study, we examined the biomechanical properties of standard trauma lag screws and iFuse-TORQ implants with a porous structure. Mechanical properties of screw fixation are dictated by outer and core diameters, length, thread design, and pitch, which all determine the pullout strength of the construct and can improve construct stability [21, 22]. The insertion and removal torques were significantly higher for the TORQ implant placed at S1 than the standard trauma lag screw implant placed at S1. Similarly, the insertion torque was significantly higher for the TORQ implant placed at S2 than the trauma lag screw placed at S2. Comparatively, the results suggest that the TORQ implants are less likely to back out especially at S1, likely due to increased removal resistance. The lack of a significant difference between the implants placed at S2 may be due in part to the lack of a roughened porous layer of the 10 mm implants where it interfaces with the SI joint. The results also suggest there are a number of meaningful correlations between insertion torque and removal torque, peak load, and steady-state load. Based on the increased diameter and roughened surface of the larger additively manufactured implants, these results are not surprising.

This rotation resistance may be further enhanced by the ongrowth, ingrowth, and through-growth that will take place over time in the additively manufactured implants’ porous and fenestrated features. Due to the larger diameters of implants, there is a theoretical increased risk of sacral foramina or pelvic cortex breakthrough. All implants, however, were successfully placed within the bony corridors above the S1 foramen for the S1 implants and between the S1 and S2 foramina for the S2 implants; this included the larger 11.5 mm implants at S1 and 10.0 mm implants at S2. This was confirmed using C-arm fluoroscopy imaging for all instrumentations and direct visualization of each specimen.

While not included in the current study, it is commonly understood that 3D printed, additively manufactured implants are not as strong or as fatigue resistant as forged and machined implants of a similar geometry [23]. One advantage of additive manufacturing, however, is the ability to design and produce complex shapes and surfaces such as those described in the current study. The mechanical testing required during implant development established that the strength and endurance limit of the current study’s additively manufactured implant was greater than that of commercially available 6.5 mm and 7.3 mm titanium alloy trauma screws (SI-BONE data on file). Also, the 5.5 mm pitch of the additively manufactured implants is greater than that of the 2.75 mm pitch of the 7.3 mm trauma screws. The increased pitch has the advantage of faster implant insertion, but the increased advancement speed might come with less tactile feel, which requires the surgeon to be aware of the fine difference between the final position and implant stripping. At the same time, increased pitch might reduce the initial amount of compression achievable across the SIJ.

Although not included in the current study, the additively manufactured implants were designed for long-term bony ongrowth, ingrowth, and through-growth via the implant’s roughened surface, porous layer, and fenestrations. This bony interaction is designed to reduce implant loosening and backout, and similar porous implants have demonstrated the ability to allow for bony ongrowth, ingrowth, and through-growth [24].

Stress relaxation models were previously shown to be more reflective of physiological conditions compared to a traditional pullout protocol [22, 25]. Peak load, time to 50% peak load, and percent load drop at steady state were comparable between both implants, highlighting a similar performance over time in terms of mechanical and viscoelastic properties of the bone–screw interface. When assessing compressive loads, there were no significant differences between the TORQ and the trauma lag screw at both sacral levels tested. About 50% of the load relaxation took place in the first two hours (average of approximately 67 min), while the load dropped 70–80% within approximately 15 h after implant insertion. This load drop may still provide enough compressive load to allow for fracture reduction, stabilization, and healing, but the percentage of remaining compression may be lower than expected by some.

The current study placed implants to the midline within the S1 and S2 sacral bodies. It is well understood that bone density within the sacrum varies greatly from the lateral cortices, through the low density ala, and into the higher density sacral bodies [26–29]. The midline placement of the current study increased the likelihood of implant bony engagement and comparable measurements between treatment groups. This is the first study to report load relaxation characteristics in iliosacral pelvic fixation. The load relaxation demonstrated in the current study is not unexpected; however, the magnitude of approximately 70–80% may be a bit surprising. In other bony anatomy, Beadle et al*.* and Gruszka et al*.* demonstrated that load relaxes considerably in scaphoid fracture repair, and Cantwell et al*.* and Migliorati et al*.* demonstrated load and torque relaxation of dental implants [18, 30–32]. In a foam model, Wähnert et al*.* reported on load relaxation of 6.5 mm cannulated lag screws and Inceoğlu et al. demonstrated cyclic load relaxation of pedicle screws [22, 25, 33]. None of the studies, however, reported load relaxation of up to 80%. This difference is due to the cancellous nature of the pelvis and the likelihood of cancellous bone stress relaxing more than the cortico-cancellous bone used in other studies. Future studies focused on the load relaxation of trans-iliac, trans-sacral screws may bear different results due to the termination of the implants in the cortico-cancellous bone of the contralateral ilium.

This study includes some limitations. Although a pilot study was performed on seven specimens, no set limit on tightening torques was found. Stripping torques were found to be mostly dependent on feel based on our cadaveric pilot but also from operating room experience. It is therefore likely that the peak load measured from our experiments was not necessarily the maximal compressive load that can theoretically be applied to the screws for fear of stripping them. It is also worth noting that screw stripping occurred with both types of implants at the S2 level. Additionally, only one surgeon inserted the implants and therefore this study did not account for surgeon variability. The load cells were also larger than surgical washers and were more likely to resist washer penetration of the lateral iliac wall than the surgical washers. Similarly, the polymeric wedge washers were quite stiff and are not believed to contribute meaningfully to the load relaxation findings. In addition, the study incorporated different implant diameters and thread profiles, which may confound the interpretation of the results. Ideally we would also like to repeat some of these experiments for bilateral trans-iliac fixation with longer implants. Lastly, the current study focused on compression relaxation loads at *t* = 0 and did not account for further changes to the loading environment following a patient’s initial movement while rising from a bed or the first assisted steps.

Achieving compression along the axis of a screw is a central surgical principle in many applications and anatomic regions of the skeleton. In the posterior pelvis, compression is desirable for several specific pathologies, including for SIJ fusion, stabilization of a traumatically disrupted SIJ, and fracture compression of vertical sacral fractures. In this study, we found that the compression load dramatically decreased by approximately 70–80% of the peak load regardless of implant type or sacral level. This suggests that pelvic implants allow for fracture reduction and stabilization, but not necessarily a high degree of compression long term.

## Data Availability

Not applicable**.**
